# Low‐dose interleukin‐2 promotes STAT‐5 phosphorylation, T_reg_ survival and CTLA‐4‐dependent function in autoimmune liver diseases

**DOI:** 10.1111/cei.12940

**Published:** 2017-03-20

**Authors:** H. C. Jeffery, L. E. Jeffery, P. Lutz, M. Corrigan, G. J. Webb, G. M. Hirschfield, D. H. Adams, Y. H. Oo

**Affiliations:** ^1^ Centre for Liver Research and National Institute for Health Research Liver Biomedical Research UnitInstitute of Immunology and Immunotherapy, University of BirminghamUK; ^2^ Institute of Metabolism and Systems Research, University of BirminghamUK; ^3^ Liver Transplant and Hepatobiliary UnitUniversity Hospital of Birmingham NHS Foundation TrustBirminghamUK

**Keywords:** autoimmune liver disease, Bcl‐2, CTLA‐4, interleukin‐2, regulatory T cells, STAT‐5

## Abstract

CD4^+^CD25^high^CD127^low^forkhead box protein 3 (FoxP3^+^) regulatory T cells (T_reg_) are essential for the maintenance of peripheral tolerance. Impaired T_reg_ function and an imbalance between effector and T_regs_ contribute to the pathogenesis of autoimmune diseases. We reported recently that the hepatic microenvironment is deficient in interleukin (IL)−2, a cytokine essential for T_reg_ survival and function. Consequently, few liver‐infiltrating T_reg_ demonstrate signal transducer and activator of transcription‐5 (STAT‐5) phosphorylation. To establish the potential of IL‐2 to enhance T_reg_ therapy, we investigated the effects of very low dose Proleukin (VLDP) on the phosphorylation of STAT‐5 and the subsequent survival and function of T_reg_ and T effector cells from the blood and livers of patients with autoimmune liver diseases. VLDP, at less than 5 IU/ml, resulted in selective phosphorylation of STAT‐5 in T_reg_ but not effector T cells or natural killer cells and associated with increased expression of cytotoxic T lymphocyte antigen‐4 (CTLA‐4), FoxP3 and CD25 and the anti‐apoptotic protein Bcl‐2 in T_reg_ with the greatest enhancement of regulatory phenotype in the effector memory T_reg_ population. VLDP also maintained expression of the liver‐homing chemokine receptor CXCR3. VLDP enhanced T_reg_ function in a CTLA‐4‐dependent manner. These findings open new avenues for future VLDP cytokine therapy alone or in combination with clinical grade T_reg_ in autoimmune liver diseases, as VLDP could not only enhance regulatory phenotype and functional property but also the survival of intrahepatic T_reg_.

## Introduction

Naturally occurring CD4^+^CD25^high^CD127^low^forkhead box protein 3 (FoxP3^+^) regulatory T cells (T_reg_) constitute 5–10% of peripheral CD4^+^ T cells and maintain peripheral self‐tolerance in rodents and humans 1, 2. Functional impairment or quantitative deficiency of T_regs_ has been described in autoimmune liver diseases (AILD) 3, 4, including those targeted at bile ducts [primary biliary cholangitis (PBC) 5, 6 or primary sclerosing cholangitis (PSC)] and hepatocytes [autoimmune hepatitis (AIH)] 3, 7, 8, 9. Current therapies for AILD are non‐curative, provide unsatisfactory control of hepatic inflammation and require long‐term immunosuppressive medications that carry unfavourable side effects. Thus, autologous T_reg_ therapy is an attractive option for the treatment of AILD that could provide long‐term immune‐regulation without daily medications and global immunosuppression.

T_reg_ survival and function is dependent upon interleukin (IL)−2 10, which is required for the maintenance of effective levels of functional T_reg_ in autoimmune diseases 11, 12, 13. The importance of IL‐2 for T_reg_ function has not been studied closely in autoimmune liver diseases. The cell surface receptor for IL‐2 (IL‐2R) is composed of three subunits, alpha (IL‐2RA, CD25), beta (IL‐2RB, CD122) and common gamma (IL‐2RG, CD132). All leucocytes express IL‐2RG constitutively. Natural killer (NK), NK T cells (NK T) and memory CD8^+^ T cells also express IL‐2RB and T_reg_ express IL‐2RA constitutively. IL‐2RA is required for high‐affinity IL‐2 binding, while IL‐2RB and IL‐2RG transduce the IL‐2 signal 14. Two major signalling pathways conduct IL‐2‐induced responses: signalling from IL‐2RB leads to the activation of the serine/threonine kinase, AKT, and to up‐regulation of anti‐apoptotic molecules such as Bcl‐2, which is required for T cell survival 15. Signalling from IL‐2RG via Janus kinase 3 (JAK3) leads to signal transducer and activator of transcription‐5 (STAT‐5) activation 16, and is needed for T cell proliferation and differentiation and expression of anti‐apoptotic molecules 17, 18. Owing to their high levels of high‐affinity CD25, T_reg_ consume IL‐2 competitively, thereby maintaining their survival and function while suppressing bystander effector cells 19, 20. Where IL‐2 availability is low, such as in the inflamed hepatic microenvironment, T_reg_ function may be compromised and may be inadequate to counteract the activated immune infiltrate.

A number of studies have indicated that treatment with IL‐2 could improve immune‐mediated diseases. In rodents, type 1 diabetes mellitus could be prevented by *in‐vivo* IL‐2 administration 21, 22. In humans, low‐dose IL‐2 therapy enhanced T_reg_ frequencies and improved outcome in graft‐*versus*‐host disease, vasculitis and type 1 diabetes 11, 23, 24, 25. We recently reported very low levels of IL‐2 in the inflamed human liver 4. Thus, we considered that clinical grade IL‐2 (Proleukin) therapy might be effective in AILD.

In this study, we examined the effect of very low dose Proleukin (VLDP) on the biology of both peripheral and intrahepatic T_reg_, focusing upon regulatory phenotype and function. Successful T_reg_ therapy in AILD would require not only enhancing T_reg_ phenotype and function, but also recruitment of peripheral T_reg_ to the inflamed autoimmune livers. We demonstrate for the first time, to our knowledge, that VLDP selectively enhances T_reg_ STAT‐5 phosphorylation and subsequently up‐regulates functional molecules cytotoxic T lymphocyte antigen‐4 (CTLA‐4), CD25, FoxP3 and T_reg_ anti‐apoptosis marker Bcl‐2 in AILD. VLDP also maintains liver homing chemokine receptor CXCR3 expression on T_reg_. Importantly, VLDP enhances the suppressive function of T_reg_ via CTLA‐4, and anti‐CTLA‐4 can block this effect. Thus, we demonstrated both phenotype and mechanistic effects of VLDP on blood and intrahepatic T_reg_ from AILD patients suggesting that VLDP therapy may enhance immune‐regulatory restoration in AILD.

## Materials and methods

### Ethics statement

Written informed consent was obtained from all subjects in this study. Local Research Ethics Committees (LREC) and the University of Birmingham approved all experimental protocols (South Birmingham LREC reference: 98 CA/5192; Walsall LREC reference: 06/Q2708/11).

### Blood and liver tissue

Venous blood, collected in ethylenediamine tetraacetic acid (EDTA) tubes, was obtained from healthy (control) individuals and individuals with AILD, including AIH, PBC and PSC. Explanted diseased liver tissue was obtained from patients undergoing liver transplantation for end‐stage AILD including PBC, PSC and AIH/PBC and AIH/PSC overlap diseases.

### Isolation of liver infiltrating leucocytes (LIL) and peripheral blood mononuclear cells (PBMC)

LIL were isolated from fresh liver tissue. Briefly, explanted liver tissue was diced into 5 mm^3^ cubes, washed with phosphate‐buffered saline (PBS) and then homogenized in a Seward stomacher 400 circulator (260 rpm, 5 min). The homogenate was filtered through fine (63 μm) mesh (John Staniar and Co., Manchester, UK) and the lymphocytes isolated by density gradient separation using Lympholyte (VH Bio, Gateshead, UK) at 800 ***g*** for 20 min. The lymphocyte layer was collected and washed with PBS. Cell viability was assessed by trypan blue exclusion. Peripheral blood lymphocytes were isolated similarly from whole blood by density gradient separation using Lympholyte.

### Culture of PBMC and LIL

PBMC and LIL were cultured in 24‐well plates at a density of 1 × 10^6^ cells/ml in RPMI‐1640 with L‐glutamine medium containing penicillin (100 IU/ml), streptomycin (100 IU/ml), additional glutamine (2 mM) (Gibco, Carlsbad, CA, USA) and 10% human AB serum (TCS Biosciences, Buckingham, UK) and supplemented with 0 or 5 IU/ml Proleukin (Aldesleukin) (Novartis, Camberley, UK).

### Surface phenotyping of freshly isolated intrahepatic and peripheral blood lymphocytes with or without Proleukin treatment

Cell phenotypes were examined by flow cytometry. Dead cells were identified by staining with the Zombie NIR^TM^ fixable viability dye (BioLegend, San Diego, CA, USA) or e506 viability dye (eBioscience, San Diego, CA, USA) prior to staining with antibodies. To analyse expression of surface antigens, cells were incubated on ice for 30 min with antibodies against CD3, CD4, CD8, CD25, CD127 and markers of interest or isotype‐matched control antibodies in 2% fetal bovine serum (FBS) (Sigma Aldrich, Poole, UK) diluted in PBS. After washing with 2% FBS (Sigma Aldrich), cells were fixed for 10 min with 3% formaldehyde solution (Sigma Aldrich). To analyse expression of intracellular proteins, cells were fixed and stained using the FoxP3/transcription factor staining set (eBioscience), according to the manufacturer's instructions. Antibodies against surface markers (CD3, CD4, CD8 CD25, CD127) were generally added together with antibodies against intracellular markers of interest during the permeabilization and intracellular staining steps. Data were acquired using a CyAN ADP flow cytometer (Dako, Glostrup, Denmark). Single fluorophore‐labelled anti‐mouse immunoglobulin (Ig)Gκ/negative control (FBS) compensation particles (BD Biosciences, Franklin Lakes, NJ, USA) were used for compensation. Data were analysed offline using FlowJo (TreeStar Inc., Ashland, OR, USA).

The anti‐human antibodies used in flow cytometric analysis of marker expression included: anti‐CD3‐phycoerythrin‐cyanin 7 (PE‐Cy7) (SK7; BD Biosciences), anti‐CD4‐peridinin chlorophyll (PerCP/Cy5.5 (RPA‐T4; eBioscience), anti‐CD4‐Viogreen (VIT4; Miltenyi Biotec), anti‐CD8‐PE‐CF594 (RPA‐T8; BD Biosciences), anti‐CD25‐BV421 (M‐A251; BD Biosciences), anti‐CD45Ra‐allophycocyanin (APC)‐Vio770 (T6D11; Miltenyi Biotec), anti‐CD127‐fluorescein isothiocyanate (FITC) (eBioRDR5; eBioscience), anti‐CCR7‐PE‐CF594 (150503; BD Biosciences), anti‐granzyme B‐PE (GB11; eBioscience), anti‐CTLA‐4‐PE (BN13; BD Biosciences), anti‐Bcl‐2‐PE (100; BioLegend), anti‐FoxP3‐APC (PCH101; eBioscience) anti‐CD39‐PE (A1; eBioscience), anti‐T cell immunoglobulin and mucin domain‐containing‐3 (TIM3)‐PE (F38‐2E2; eBioscience), anti‐OX40‐PE (ACT35; BD Biosciences), anti‐CD69‐PE (FN50; Miltenyi Biotec), anti‐2B4‐PE (REA112; Miltenyi Biotec), anti‐CD73‐APC (AD2; eBioscience), anti‐CD137‐APC (4B4‐1; Miltenyi Biotec), anti‐glucocorticoid‐induced tumour necrosis factor receptor (GITR)‐APC (DT5D3; Miltenyi Biotec), anti‐lymphocyte‐activation gene 3 (LAG3)‐APC (3DS223H; eBioscience) and anti‐PD‐1‐APC (PD1.3.1.3; Miltenyi Biotec).

### Analysis of STAT‐5 phosphorylation in response to IL‐2

To examine responsiveness of PBMC or LIL to IL‐2, cells in RPMI were stained with anti‐CD127‐FITC (eBioRDR5; eBioscience), anti‐CD20‐Viogreen (LT20; Miltenyi Biotec) and anti‐CD56‐pacific blue (HCD56; Biolegend) for 10 min at room temperature. Cells were then stimulated for 10 min at 37°C with Proleukin (0‐1000 IU/ml). Cells were fixed and permeabilized with BD Biosciences Phosflow buffers I and III according to the manufacturer's instructions then stained for 1 h at room temperature in 2% FBS (Sigma) with anti‐pSTAT‐5 (Y694)‐AlexaFluor 647 (47/STAT‐5), anti‐CD3‐PeCy7 (SK7), anti‐CD8‐PE‐CF594 (RPA‐T8) (all from BD Biosciences), anti‐CD4‐PerCPCy5.5 (RPA‐T4; eBioscience) and anti‐CD25‐PE (3G10; Miltenyi Biotec). All data were acquired using a CyAn ADP (Dako) flow cytometer and analysed using FlowJo (Tree Star) software.

### Analysis of suppression of autologous T responder cell division by T_reg_ in response to IL‐2

CD4^+^CD25^+^CD127^–^ T_reg_ and CD4^+^CD25^–^CD127^+^ responder T cells were isolated by flow sorting following prior enrichment of total CD4^+^ T cells from PBMC using magnetic negative selection (Biolegend). T_reg_ were cultured overnight with or without Proleukin (5 IU/ml). Responder T cells were labelled with cell trace violet (molecular probes; Thermofisher, Waltham, MA, USA) and cultured overnight without stimulation. Dendritic cells (DC) were derived from monocytes that were isolated from healthy donor PBMCs by magnetic negative selection (StemCell Technologies, Vancouver, Canada) and cultured for 5–7 days in IL‐4 (500 IU/ml; Miltenyi Biotec) and granulocyte–macrophage colony‐stimulating factor (GM‐CSF) (800 IU/ml; Berlex Laboratories, Richmond, CA, USA). DCs and T_reg_ were washed to remove cytokines and co‐cultured for 5 days with responder T cells in the presence of 0·5 μg/ml anti‐CD3 (clone OKT3; Biolegend) with or without 40 μg/ml anti‐CTLA‐4. Division of responder T cells under 50 μg/ml CTLA‐4‐Ig (abatacept) was also monitored. Cells were cultured at a ratio of 1 DC : 20 T cells with a 1 T_reg _: 2·5 responder T cell ratio. At 5 days, cell trace violet dilution was measured by flow cytometry and the statistics percentage division and division index (the average number of cell divisions undergone by a cell in the original population) calculated using the FlowJo proliferation analysis platform.

### Statistical analysis

Statistical significance between two variables across multiple subsets was tested by two‐way two‐tailed analysis of variance (anova) with Bonferroni's *post‐hoc* analysis. Significance between two populations was tested by paired *t*‐test and between multiple treatments by one way anova with Bonferroni's *post‐hoc* analysis. Analysis and graphical representation was performed using GraphPad Prism version 5 (GraphPad software, San Diego, CA, USA).

## Results

### Phosphorylation of STAT‐5 occurs selectively in T_reg_ at very low doses of Proleukin in normal blood

**Figure 1:**
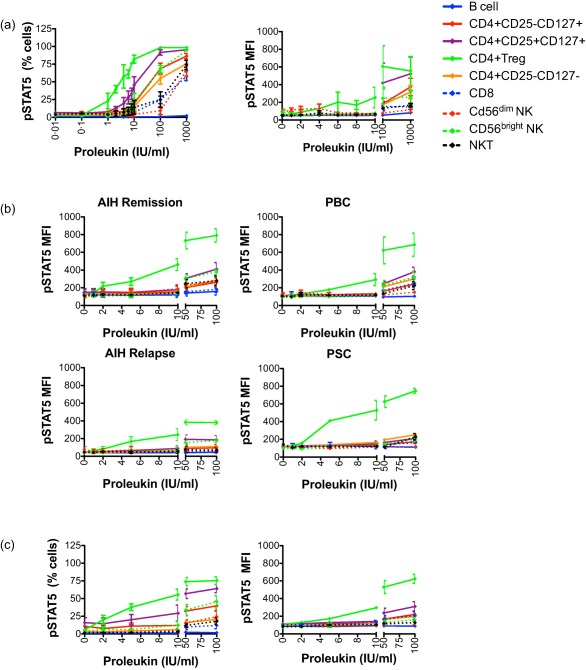
Very low dose interleukin (IL)−2 up‐regulates phosphorylated signal transducer and activator of transcription‐5 (pSTAT‐5) selectively in peripheral and liver‐infiltrating regulatory T cells (T_reg_) from patients with autoimmune liver disease. Peripheral blood mononuclear cells (PBMCs) from control bloods (a, *n* = 3) or autoimmune liver disease patient bloods [b, autoimmune hepatitis (AIH) remission, *n* = 5; AIH relapse, *n *= 4; primary biliary cholangitis (PBC), *n* = 3; and primary sclerosing cholangitis (PSC), *n* = 3] and liver‐infiltrating lymphocytes from explanted livers of patients with autoimmune liver disease (c, *n* = 4) were stimulated for 10 min with IL‐2 (Proleukin) at doses in the range 0–1000 IU/ml and the expression of p(Y694) STAT‐5 determined by flow cytometry for each leucocyte population. Graphs summarize pSTAT‐5 expression, quoted as percentage of positive cells or median fluorescence intensity (MFI). Data are mean ± standard error of the mean (s.e.m.).

To analyse the responsiveness of different leucocyte populations to IL‐2 (Proleukin) and identify a dose that would phosphorylate STAT‐5 selectively in T_reg_, we administered Proleukin at doses of between 0 and 1000 IU/ml to PBMC from healthy control bloods and examined phosphorylation of STAT‐5 in T_reg_ and other leucocyte populations: (Fig. 1a and Supporting information, Fig. 1). With the exception of B cells, all cell types responded to Proleukin at 10 IU/ml and above (Fig. 1a). However, at 1 IU/ml, 25% of T_reg_ showed measurable pSTAT‐5 expression, while there was no up‐regulation in pSTAT‐5 in other subsets. More than 75% of T_reg_ demonstrated pSTAT‐5 induction at 10 IU/ml; however, this dose also led to phosphorylation of STAT‐5 in other CD4^+^ T cells and CD56^bright^ NK cells. Nevertheless, this pSTAT‐5 induction in non‐T_regs_ at this concentration was barely above baseline, as indicated by pSTAT‐5 median fluorescence intensity (MFI) (Fig. 1a). Most importantly, at 2–4 IU/ml, T_reg_ pSTAT‐5 MFI was 2·3–3·6‐fold above baseline, but there was no increase in pSTAT‐5 MFI for other subsets (Fig. 1a). Thus, we observed that with a very low dose of Proleukin (VLDP) (< 5 IU/ml), selective phosphorylation of STAT‐5 in T_reg_ could be achieved. The characteristics of the control cohort used in these and subsequent phenotyping studies in this paper are given in Table 1.

**Table 1:** Demographic, laboratory, histology and treatment details of patients in the peripheral blood cohort

Disease	Age (years)	Sex	BR	AST	ALT	ALP	Na+	CR	IgM	IgG	INR	UKELD	Histology	Treatment
AIH F	28	M	19	78	23	65	139	68	0·6	17	1·2	49	Moderate hepatitis, established cirrhosis	Azathioprine + tacrolimus + prednisolone
AIH F	61	F	10	129	125	120	139	71	0·5	25	1·2	47	Moderate‐ severe activity, F4	Mycophenolate
AIH F	57	F	15	76	53	114	140	61	1·4	18	1·1	48	Moderate hepatitis, F3	Azathioprine + prednisolone
AIH F	42	M	37	68	120	104	142	86	1·3	17	1·0	49	Moderate hepatitis, established cirrhosis	Azathioprine, prednisolone
AIH F	64	M	7	72	127	93	141	87	0·7	17	1·0	45	Moderate lobular hepatitis, F2	Mycophenolate + prednisolone
AIH F	24	M	59	167	253	120	138	49	0·7	21	1·2	53	Moderate hepatitis, F3	Mycophenolate + prednisolone
AIH R	58	M	19	42	30	82	142	86	1	16	0·9	47	Moderate lobular inflammatory activity, F2	Azathioprine
AIH R	46	F	4	17	8	76	137	67	1·2	13	1·0	44	Mild hepatitis, F2	Azathioprine
AIH R	51	F	11	26	21	58	143	72	1	10	1·0	45	Quiescent AIH, cirrhotic	None
AIH R	58	F	15	39	40	90	144	58	0·7	12	1·1	45	Quiescent AIH, F1	Azathioprine + prednisolone
AIH R	45	F	10	16	11	86	142	67	0·9	9	1·0	45	Mild hepatitis, F1	Azathioprine
AIH R	35	F	14	22	19	75	139	58	1	11	1·1	48	Moderate hepatitis, F3	Azathioprine
AIH R	54	F	7	21	28	76	143	66	1·2	9	1·0	43	Moderate hepatitis, F3	Mycophenolate + prednisolone
AIH R	45	F	13	43	36	84	138	64	1	20	1·0	47	Mild hepatitis, F2	Azathioprine
AIH R	21	F	17	30	35	70	143	63	1	8	1·0	46	Mild hepatitis, Established cirrhosis	Azathioprine + prednisolone
AIH R	57	F	3	25	28	65	142	82	0·8	12	1·0	42	Mild hepatitis, F2	Mycophenolate + prednisolone
AIH R	33	F	7	28	24	46	139	65	0·6	11	1·0	46	Mild lobular, hepatitis, F2	Mycophenolate + prednisolone
AIH R	51	F	8	33	33	93	140	60	1·3	12	1·0	46	Mild hepatitis, F1	Mycophenolate + prednisolone
AIH R	36	M	11	18	22	67	141	58	0·8	14	1·2	45	Mild hepatitis, established cirrhosis	Azathioprine
AIH R	21	F	11	17	19	67	141	61	0·8	8	1·0	46	Mild hepatitis, F1	Mercaptopurine
PBC	72	F	8	36	34	210	144	105	2·9					
PBC	63	F	5	32	20	160	141	83	3·5	9·37	0·9	43	Non‐cirrhotic	Ursodeoxycholic acid
PBC	51	F	39	113	76	184	142	93	5·6	16·81	1·1	49	Non‐cirrhotic	Ursodeoxycholic acid
PBC	47	M	13	n.a.	32	220	140	88	6·6	24·88	1·0	47	Chronic biliary disease, paucity of bile ducts, cirrhosis	Ursodeoxycholic acid
PBC	63	F	5	32	20	160	141	83	3·5	9·37	0·9	43	Non‐cirrhotic	UDCA
PBC	51	F	39	113	76	184	142	93	5·6	16·81	1·1	49	Non cirrhotic	UDCA
PBC	47	F	13	29	21	150	142	52	1·71	10·64	1·1	46	2007 ‐ PBC with FNH; no cirrhosis	UDCA
PBC	58	F	11	n.a.	88	653	138	66	7·15	23·63	1·0	47	n.a.	UDCA
PBC	41	F	5	20	23	74	145	68	1·31	12·75	1·1	42	n.a.	UDCA
PBC	47	F	32	112	129	446	139	57	7·78	13·32	1·0	50	n.a.	UDCA
PSC	54	F	4	14	19	297	142	78	0·6	9	0·8	42	PSC, F2	UDCA
PSC	43	M	70	151	68	575	139	60	0·8	25	1·2	54	PSC cirrhotic	UDCA
PSC	77	M	3	21	10	64	136	118	0·6	10	0·9	45	PSC, F2	UDCA
Control	45	M	8	12	18	n.a.	133	69	n.a.	n.a.	0·9	49	n.a.	None
Control	56	F	4	8	20	n.a.	140	74	n.a.	17·07	1·0	43	n.a.	None
Control	60	M	10	41	55	n.a.	145	105	n.a.	15·75	1·1	44	n.a.	None
Control	49	M	18	16	13	n.a.	141	85	n.a.	10·11	1·1	48	n.a.	None
Control	60	M	3	12	16	n.a.	139	76	n.a.	N/A	n.a.	n.a.	n.a.	None
Control	67	F	9	33	28	n.a.	140	64	n.a.	n.a.	1·1	46	n.a.	None
Control	42	M	6	24	21	n.a.	135	72	n.a.	n.a.	n.a.	n.a.	n.a.	None
Control	76	M	6	32	27	n.a.	143	79	n.a.	12·71	n.a.	n.a.	n.a.	None
Control	41	M	63	26	12	n.a.	140	93	n.a.	9·19	n.a.	n.a.	n.a.	None

AIH R = autoimmune hepatitis in remission; AIH F = autoimmune hepatitis in flare up/relapse; PSC = primary sclerosing cholangitis; PBC = primary biliary cholangitis; AST = aspartate transaminase; ALT = alanine transaminase; ALP = alkaline phosphatase BR = bilirubin; Na+ = sodium; CR = creatinine; UKELD = United Kingdom End Stage Liver Disease Scoring; UDCA = ursodeoxycholic acid; n.a. = not available.

### Very low doses of Proleukin induce selective phosphorylation of STAT‐5 in intrahepatic and peripheral blood T_reg_ of patients with autoimmune liver diseases

PBMC from AIH, PBC and PSC patients were treated with Proleukin in the range of 0–100 IU/ml. Similar to controls, T_reg_ of AIH patients [both remission and flare‐up (relapse)], PBC patients and PSC patients demonstrated a selective pSTAT‐5 induction at doses below 10 IU/ml, especially at 5 IU/ml (Fig. 1b and Supporting information, Fig. 2). We then treated human LIL isolated from explanted autoimmune liver disease tissues with 0–100 IU/ml Proleukin, and again selective pSTAT‐5 enhanced induction was seen in T_reg_ at doses below 10 IU/ml (Fig. 1c). Descriptions of all AILD blood and liver explant donors whose PBMC and LIL were used in these and subsequent studies in this paper to address the effect of IL‐2 on phenotype are given in Tables 1 and 2 [blood donors were: AIH = 20 (aged 44·4 ± 13·8 years, disease in remission = 14, disease in flare up/relapse = 6); PSC = 3 (aged 58·0 ± 17·3 years); PBC = 10 (aged 54·0 ± 9·6 years); control = 9 (aged 55·1 ± 11·9 years)]. Liver donors were: PBC = 5 (aged 59·4 ± 6·4 years); PSC = 2 (aged 51·5 ± 23·3 years); AIH overlap = 2 (aged 38·0 ± 24·0 years), PBC/AIH overlap = 1; and PSC/AIH overlap = 1).

**Table 2:** Demographic, histology and treatment details of patients in the liver explant cohort

Disease	Age (years)	Sex	Explant history	Treatment before transplantation
PBC	51	M	Moderate fibrosis	Ursodeoxycholic acid
PBC	63	F	Established cirrhosis	Ursodeoxycholic acid
PBC	65	F	Established cirrhosis	Ursodeoxycholic acid
PBC	64	F	Established cirrhosis	Ursodeoxycholic acid
PBC	54	F	Established cirrhosis	Ursodeoxycholic acid
PSC	68	M	Established cirrhosis	
PSC	35	M	Established cirrhosis	
PBC/AIH overlap	55	F	Established cirrhosis	Ursodeoxycholic acid
PSC/AIH overlap	21	F	Established cirrhosis	

AIH = autoimmune hepatitis; PBC = primary biliary cholangitis; PSC = primary sclerosing cholangitis.

### Very low doses of Proleukin induce T_reg_ functional markers CTLA‐4, CD25 and FoxP3 selectively in both blood and intrahepatic T_reg_

**Figure 2:**
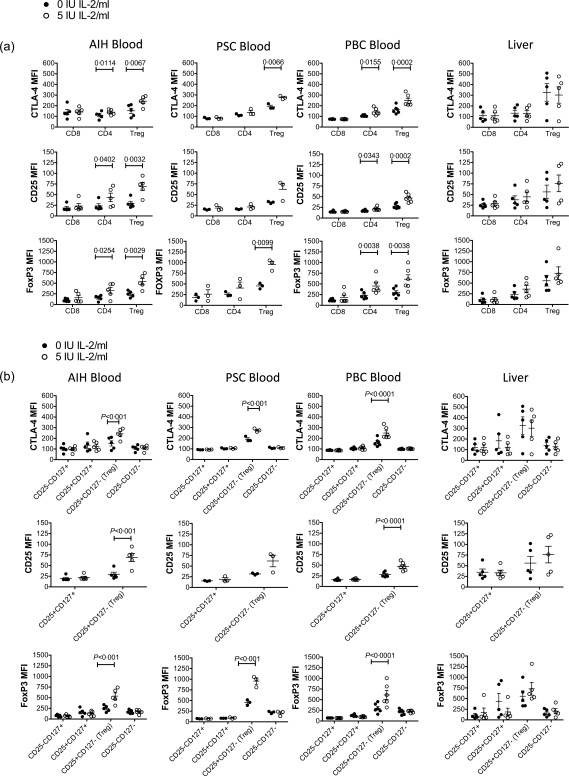
Effect of very low dose interleukin (IL)−2 on expression of IL‐2‐regulated regulatory T cells (T_reg_) functional markers CD25, cytotoxic T lymphocyte antigen‐4 (CTLA‐4) and forkhead box protein 3 (FoxP3) by T_reg_ cells from blood and liver. Peripheral blood mononuclear cells (PBMCs) from patients with autoimmune hepatitis (AIH) (*n* = 5), primary sclerosing cholangitis (PSC), (*n* = 3) and primary biliary cholangitis (PBC), *n* = 6) and liver‐infiltrating leucocytes from autoimmune liver diseases (AILD) livers were exposed to 0 or 5 IU/ml IL‐2 (Proleukin) for 18 h and the median fluorescence intensity of CD25, CTLA‐4 and FoxP3 examined by flow cytometry for (a) CD4, CD8 and T_reg_ cells and (b) CD4 subsets defined by CD25 *versus* CD127 expression. Data are mean ± standard error of the mean (s.e.m.). Significant effects of IL‐2 analysed by paired *t*‐tests (a) and two‐way analysis of variance (anova) with Bonferroni's *post‐hoc* test (b) are shown.

Having found 5 IU/ml Proleukin by dose titration to be the optimal concentration to induce a selective STAT‐5 response in blood and liver T_reg_ of AILD patients, we determined the effect of 5 IU/ml Proleukin on T cell phenotype and function. PBMC and LIL from AILD patients were exposed to 0 IU/ml or 5 IU/ml Proleukin for 18 h and effects on markers of T cell activation and T_reg_ function by T cell subsets were assessed; 5 IU/ml Proleukin increased significantly the levels of CD25, CTLA‐4 and FoxP3 on peripheral blood T_reg_ of AILD patients (Fig. 2a and Supporting information, Fig. 3). In liver, basal expressions of CTLA‐4, CD25 and FoxP3 on T_reg_ were higher than in blood and similar to those observed in blood following Proleukin exposure (Fig. 2a). There were clear trends towards increased expression of the T_reg_ markers CD25 and FoxP3 with VLDP in liver T_reg_ from PBC, PSC and AIH, supporting potentiation of T_reg_ in the liver in settings of the AILDs (Fig. 2a and Supporting information, Fig. 3). These observations are consistent with and also reflective of upstream pSTAT‐5 induction by VLDP in liver T_reg_ (Fig. 1c). Frequencies of expression of CD25, CTLA‐4 and FoxP3 were largely not altered in blood or liver T_reg_ by VLDP (Supporting information, Fig. 4).

Because we noticed that 18 h exposure to Proleukin led to STAT‐5 phosphorylation in CD25^+^CD127^+^CD4^+^ T cells that mirrored that in T_reg_ (CD25^+^CD127^–^CD4^+^) (both frequency and MFI) (Supporting information, Fig. 5), we evaluated the effect of 18‐h VLDP on the expression of CD25, CTLA‐4 and FoxP3 by the CD25^+^CD127^+^CD4^+^ T effector cell population in the blood of AILD patients (Fig. 2b). Importantly, despite phosphorylation of STAT‐5, this subset, unlike T_reg_, did not up‐regulate CD25, CTLA4 or FoxP3 (Fig. 2b).

**Figure 3:**
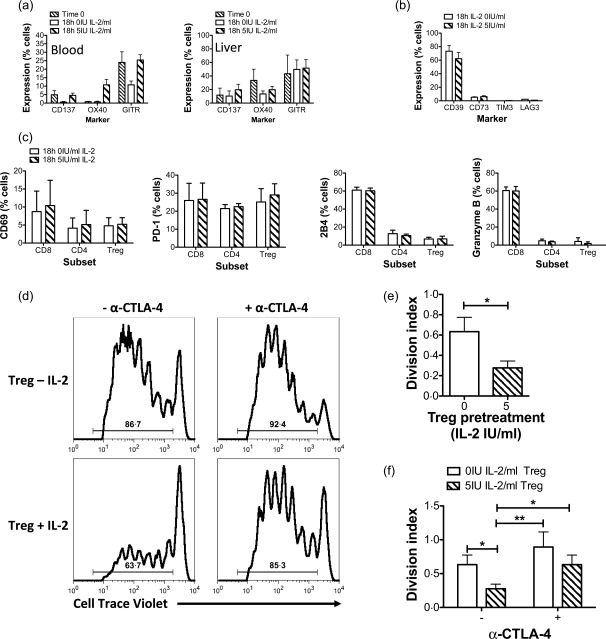
(a–c) Effect of very low dose interleukin (IL)−2 on regulatory T cells (T_reg_) and T effector functional and activation phenotypes. Peripheral blood mononuclear cells (PBMCs) from patients with autoimmune hepatitis (AIH) and liver infiltrating leucocytes from autoimmune liver diseases (AILD) livers were exposed to 0 or 5 IU/ml IL‐2 (Proleukin) for 18 h and the expression of functional and activation markers by T cell subsets analysed by flow cytometry. (a) Expression of tumour necrosis factor (TNF) receptor superfamily members by blood and liver T_reg_. (b) Expression of CD39, CD73, T cell immunoglobulin and mucin domain‐containing‐3 (TIM3) and lymphocyte‐activation gene 3 (LAG3) by blood T_reg_. (C) Expression of CD69, programed death 1 (PD‐1), 2B4 and granzyme B by blood CD4, CD8 and T_reg_. Data are mean ± standard error of the mean (s.e.m.) for 2–6 donors. (d–f) Very low dose IL‐2 increases T_reg_ suppressive ability in a mechanism involving cytotoxic T lymphocyte antigen‐4 (CTLA‐4). CD4^+^CD25^+^CD127^–^ T_reg_ and autologous CD4^+^CD25^–^ T responder cells were isolated from PBMC of control individuals. T responders were labelled with cell trace violet and following overnight exposure of T_reg_ to 0 or 5 IU/ml Proleukin were co‐cultured with the T_reg_ in the presence of anti‐CD3 and dendritic cells, with or without CTLA‐4 blockade. Cell trace violet dilution indicating T responder cell division was analysed by flow cytometry at 5 days. (d) Representative flow cytometry histograms of T responder division showing percentage division. (e) Division index summary data (*n* = 3) of T responders in the presence of T_reg_ pretreated with 0 or 5 IU/ml Proleukin. (f) Division index summary data (*n* = 3) of T responders in the presence of T_reg_ pretreated with 0 or 5 IU/ml Proleukin with or without anti‐CTLA‐4. Data are mean ± standard error of the mean (s.e.m.). Significant effects of IL‐2 and CTLA‐4 blockade analysed by paired *t*‐tests (e) and one‐way analysis of variance (anova) with Bonferroni *post‐hoc* test (f) are shown.

We then investigated the effect of VLDP on the expression by peripheral and liver‐infiltrating T_reg_ of tumour necrosis factor (TNF) receptor superfamily members CD137, OX40 and GITR. Culture in 5 IU/ml Proleukin helped to maintain expression frequencies of CD137 and GITR on peripheral T_reg_ over 18 h and promoted an increase in the frequency of OX40 expressing peripheral T_reg_ (Fig. 3a and Supporting information, Fig. 3). Exposure to VLDP did not influence the frequency of expression of these markers on liver‐infiltrating T_reg_ (Fig. 3a). We also investigated the impact of VLDP on T_reg_‐associated surface markers, including CD39, CD73 and LAG3. Proleukin 5 IU/ml had no effect on the expression frequency of any of these molecules (Fig. 3b and Supporting information, Fig. 3): high expression of CD39 was maintained, CD73 remained low‐expressed and TIM3 and LAG3, which were not detected at baseline, were not induced by Proleukin treatment on T_reg_. Also, VLDP did not alter expression of the activation marker, CD69 or the immune checkpoint and programmed cell death receptor‐1 (PD‐1) on CD4^+^ or CD8^+^ T cells or T_reg_ (Fig. 3c and Supporting information, Fig. 3). Consistent with a lack of pSTAT‐5 induction in CD8^+^ T cells under VLDP, 5 IU/ml Proleukin did not affect expression of granzyme B or activation marker 2B4 by CD8^+^ T cells (Fig. 3c and Supporting information, Fig. 3).

### Very low dose Proleukin enhances T_reg_ suppressive potential in a CTLA‐4‐dependent manner across all autoimmune liver diseases

Having established that VLDP 5 IU/ml enhances T_reg_ expression of regulatory functional molecules we sought to verify that these changes in phenotype associate with improvements in T_reg_ function. We tested whether exposure to VLDP could increase the ability of T_reg_ to suppress the division of autologous CD25^–^CD4^+^ T effector cells. We used a system of anti‐CD3 together with monocyte‐derived DC to supply co‐stimulatory ligands. This system was chosen over conventional anti‐CD3/CD28 bead activation of T cell proliferation in order that the functional impact of antigen‐presenting cell–T_reg_ contact‐dependent mechanisms of suppression such as involving CTLA‐4 might be studied. Pre‐treatment of T_reg_ with Proleukin reduced significantly the division of T responder cells from control individuals cultured 2·5 : 1 with autologous T_reg_ (Fig. 3d,e). This elevated suppressive potential of VLDP‐treated T_reg_ was overcome in the presence of anti‐CTLA‐4 (Fig. 3d,f). Consistent with our observations that CTLA‐4‐Ig can reduce T responder division (Supporting information, Fig. 6a), anti‐CTLA‐4 also tended to increase T responder division in the presence of untreated T_reg_ (Fig. 3f). We observed the same trends of improved T_reg_ function involving contribution from CTLA‐4 with VLDP treatment of T_reg_ in both cases of AILD patients tested (Supporting information, Fig. 6b).

### Very low dose Proleukin treatment does not alter liver homing CXCR3 expression by peripheral blood T_reg_ in autoimmune liver diseases

**Figure 4:**
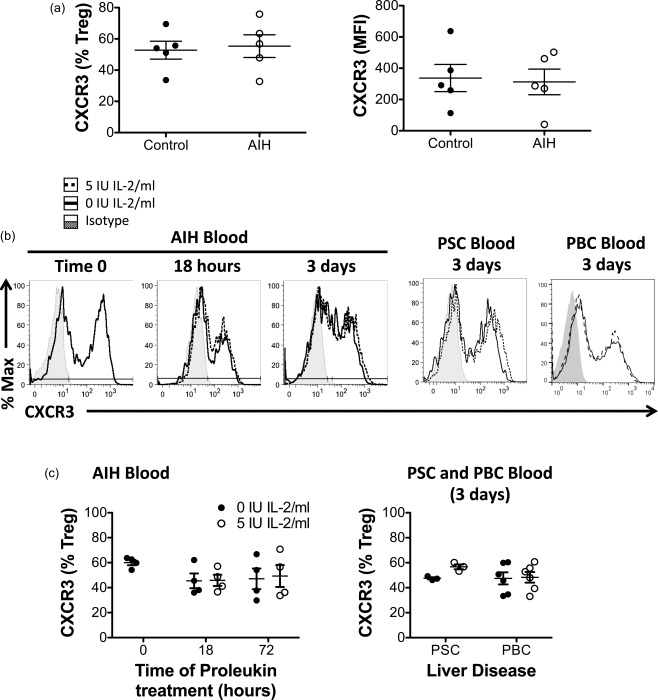
Very low dose interleukin (IL)−2 does not down‐regulate liver‐homing CXCR3 receptor on regulatory T cells (T_reg_). (a) Peripheral blood mononuclear cells (PBMCs) were isolated from controls and autoimmune hepatitis (AIH) patients and T_reg_ phenotyped for CXCR3 by flow cytometry *ex vivo*. (b,c) CXCR3 expression was measured in PBMC T_reg_ from patients with AIH, primary sclerosing cholangitis (PSC) or primary biliary cholangitis (PBC) *ex vivo* and after culture for 18 h and/or 3 days in 5 IU/ml IL‐2 (Proleukin). Expression of CXCR3 by CD4^+^CD25^+^CD127^–^ T_reg_ of one representative donor from each disease cohort is shown in (b) and expression summarized for AIH, PSC donors and PBC donors in (c). Data are mean ± standard error of the mean (s.e.m.).

Regulatory T cell homing to the site of liver inflammation is crucial for the success of T_reg_ therapies, thus we examined the crucial liver‐homing chemokine receptor, CXCR3, on blood T_reg_ of autoimmune liver disease patients. *De‐novo* expression levels (MFI and frequency) of CXCR3 were similar in patients with AIH compared to controls (Fig. 4a). Importantly, VLDP 5 IU/ml did not have any impact upon CXCR3 expression in AIH patients over 18 h or up to 3 days (Fig. 4b,c). Peripheral T_reg_ from PSC and PBC patients also showed no change in their CXCR3 expression with sustained culture in VLDP (Fig. 4b,d), thus VLDP therapy may be applicable for all three autoimmune liver diseases without impairing the recruitment capacity of T_reg_.

### Very low dose Proleukin treatment increases total T_reg_ frequencies and promotes the strongest T_reg_ suppressive phenotype in the CD45RA^–^CCR7^–^ population of T_reg_

**Figure 5:**
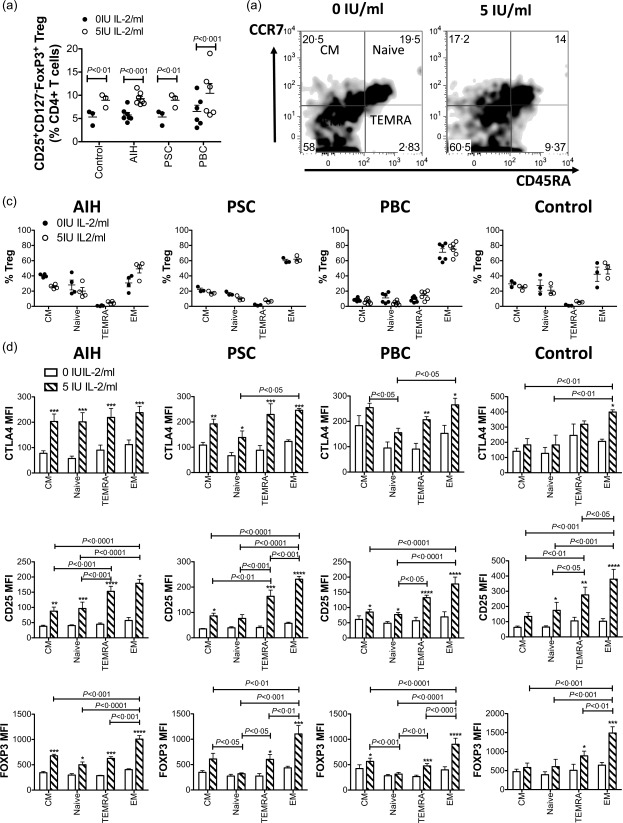
Low dose Proleukin treatment increases total regulatory T cell (T_reg_) frequencies and promotes the strongest T_reg_ phenotype in the CD45Ra^–^CCR7^–^ effector memory population of T_reg_. (a) Peripheral blood mononuclear cells (PBMCs) from controls, autoimmune hepatitis (AIH), primary sclerosing cholangitis (PSC) and primary biliary cholangitis (PBC) patients were exposed to 0 or 5 IU/ml IL‐2 (Proleukin) for 3 days and the frequency of CD4^+^CD25^+^CD127^–^forkhead box protein 3 (FoxP3)^+^ T_reg_ assessed by flow cytometry. Significant effects of IL‐2 were analysed by two‐way analysis of variance (anova) with Bonferroni's *post‐hoc* analysis. Frequencies of memory and naive subsets of CD4^+^CD25^+^CD127^–^FoxP3^+^ T_reg_ as defined by CD45Ra and CCR7 expression were also determined at 3 days by flow cytometry. (b) Representative flow cytometry density plot of CCR7 *versus* CD45Ra for one PSC donor after 3 days of culture in 0 or 5 IU/ml Proleukin and summary data for all donors (c). Significant effects of IL‐2 on the frequencies of the different memory/naive subsets were compared by two‐way anova with Bonferroni's *post‐hoc* analysis, but no significant differences were identified. (D) Expression of cytotoxic T lymphocyte antigen‐4 (CTLA‐4), CD25 and FoxP3 by each memory or naive subset of T_reg_. Data are mean ± standard error of the mean (s.e.m.) (control (*n* = 3), AIH (*n* = 4), PSC (*n* = 3) and PBC (*n* = 6), central memory (CM) (CD45Ra^–^CCR7^+^); naive (CD45Ra^+^CCR7^+^); terminally differentiated effector memory (TEMRA) (CD45Ra^+^CCR7^–^); effector memory (EM) (CD45Ra^–^CCR7^–^). Effects of 5 IU/ml Proleukin on the expression of these markers by each subset were analysed by two‐way anova with Bonferroni's *post‐hoc* tests. Stars indicate where there was a significant effect of IL‐2 upon the expression of the marker by the subset (*P < 0·05; **P < 0·01; ***P < 0·001; ****P < 0·0001). Braces with stated *P*‐values indicate significant differences in expression by the subsets under 5 IU/ml Proleukin conditions. With the exception of CTLA‐4 expression by naive *versus* EM cells in PSC patients, or of FoxP3 expression by CN *versus* naive, CM *versus* TEMRA or TEMRA *versus* effector memory in PBC patients, there were no significant differences between the subsets for expression of any marker in the absence of IL‐2 treatment.

We noted that the CD25^+^CD127^–^FoxP3^+^ T_reg_ population, as a proportion of total CD4^+^ T cells, increased significantly with culture in VLDP up to 3 days in AILD patients and controls (Fig. 5a). No differences in frequency were seen between any of the patient or control groups at either condition. The naive (CD45RA^+^CCR7^+^) subset of T_reg_ has been reported to maintain the T_reg_‐specific demethylated region (TSDR) following expansion, making them an attractive option for T_reg_ cell immunotherapy 26, 27; thus, we evaluated the effect of 3 days VLDP on frequencies of each of the maturation subsets of CD25^+^CD127^–^FoxP3^+^ T_reg_ defined by CD45RA and CCR7 expression 28. VLDP did not alter the proportions of the maturation subsets in any AILD patient cohorts (AIH, PSC, PBC) or controls (Fig. 5b,c). None the less, as before, VLDP increased significantly the expression of IL‐2 regulated markers CTLA‐4, CD25 and FoxP3 on T_reg_ from all subsets. Overall, across AIH, PSC, PBC and control, the effector memory (CD45RA^–^CCR7^–^) subset of CD25^+^CD127^–^FoxP3^+^ T_reg_ cells showed the greatest induction of T_reg_ functional markers with VLDP treatment (Fig. 5d).

### Liver‐infiltrated T_reg_ are restricted predominantly to the CD45RA^–^CCR7^–^ population

**Figure 6:**
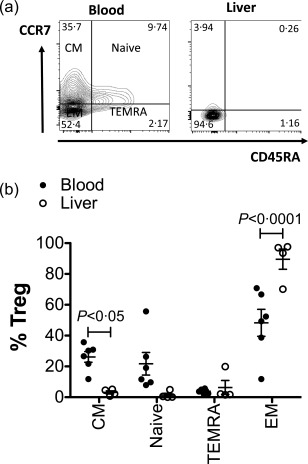
Comparison of the frequencies of memory and naive regulatory T cell (T_reg_) populations in blood and liver. (a) Frequencies of memory and naive subsets of CD4^+^CD25^+^CD127^–^ T_reg_ as defined by CD45Ra and CCR7 expression were determined by flow cytometry for peripheral blood mononuclear cells (PBMCs) and liver infiltrating lymphocytes from patients with autoimmune liver diseases (AILD). (a) Representative flow cytometry density plot for expression of CD45Ra and CCR7 by blood and liver infiltrating T_reg_ showing the four subsets including: central memory (CM) (CD45Ra^–^CCR7^+^); naive (CD45Ra^+^CCR7^+^); terminally differentiated effector memory (TEMRA) (CD45Ra^+^CCR7^–^); effector memory (EM) (CD45Ra^–^CCR7^–^). (b) Summary frequencies for each memory and naive subset in blood [*n* = 6 donors with autoimmune hepatitis (AIH)] and liver [*n* = 4 donors with AILDs including primary sclerosing cholangitis (PSC) and primary biliary cholangitis (PBC)]. Data are mean ± standard error of the mean (s.e.m.). Significance was tested by non‐matched two‐way analysis of variance (anova) and showed significant interaction for subset *versus* tissue *P* < 0·0001. Bonferroni's *post‐hoc* tests identified significant differences between blood and liver in the frequencies of CM and EM cells, as indicated. There was no significant effect of tissue on subset distribution but there was a significant effect of subset *P* < 0·0001.

Blood CD25^+^CD127^–^FoxP3^+^ T_reg_ subsets in AILD patients are comprised of approximately 60% effector memory (CD45RA^–^CCR7^–^), 15–20% central memory (CD45RA^–^CCR7^+^) and 15–20% naive (CD45RA^+^CCR7^+^) populations. Because VLDP increased significantly the expression of T_reg_ functional markers CTLA‐4, CD25 and FoxP3, especially in the CD45RA^–^CCR7^–^ subset of T_reg_, we investigated the frequency of these subsets in the inflamed human liver. We found that the effector memory CD45RA^–^CCR7^–^ subset of CD25^+^CD127^–^FoxP3^+^ T_reg_ population is increased significantly (95%) in the inflamed human livers compared to peripheral blood (60%) (Fig. 6a,b).

### Very low dose Proleukin up‐regulates expression of the anti‐apoptotic molecule Bcl‐2 on T_reg_ compared to effector T cells

**Figure 7:**
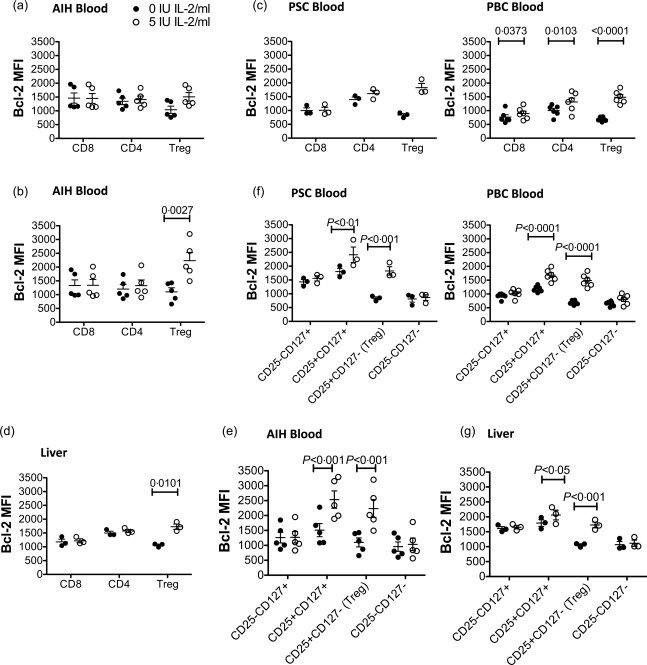
Very low dose interleukin (IL)‐2 up‐regulates Bcl‐2 expression in regulatory T cells (T_reg_) from blood and liver. Peripheral blood mononuclear cells (PBMCs) from autoimmune hepatitis (AIH) patients (a,b,e), primary sclerosing cholangitis (PSC) patients (c,f), primary biliary cholangitis (PBC) patients (c,f) and liver‐infiltrating lymphocytes from autoimmune liver diseases (AILD) livers (d,g) were exposed to 0 or 5 IU/ml IL‐2 (Proleukin) for 18 h (a) or 3 days (b–g) and the median fluorescence intensity (MFI) of Bcl‐2 on CD4, CD8 and T_reg_ examined by flow cytometry (a–d). (E–G) Day 3 Bcl‐2 expression by blood (e,f) and liver (g) CD4^+^ T cell subsets defined by CD25 *versus* CD127 expression. Data are mean ± standard error of the mean (s.e.m.). Significant effects of IL‐2 were assessed by paired *t*‐tests (a–d) and two‐way analysis of variance (anova) (e–g).

The anti‐apoptotic protein Bcl‐2 is regulated differentially in effector T cells *versus* T_reg_ 29. To assess whether VLDP alters Bcl‐2 expression, lymphocytes from AILD patients and controls were treated with 5 IU/ml Proleukin for 18 h or 3 days and their Bcl‐2 expression was evaluated. While expression by total CD4^+^ and CD8^+^ T cells was not altered up to 3 days, VLDP tended to increase blood T_reg_ expression of Bcl‐2 by 18 h (Fig. 7a). By 3 days, a significant twofold up‐regulation of Bcl‐2 expression in T_reg_ of patients with AIH was seen (Fig. 7b), and the same trend was observed in PSC and PBC (Fig. 7c). We also verified the effect of VLDP on Bcl‐2 in AILD liver T_reg_ at 3 days. Consistent with the response found in blood, we observed selective up‐regulation of Bcl‐2 in liver T_reg_ (Fig. 7d), which were of PBC and PSC disease backgrounds. In view of the STAT‐5 response by the CD4^+^CD25^+^CD127^+^ subset with sustained exposure to VLDP, we analysed the effect of VLDP on Bcl‐2 expression by each CD4^+^ T cell subset, defined by CD25 *versus* CD127 expression at 3 days in blood (Fig. 7e,f) and liver (Fig. 7g), and saw that in blood the CD4^+^CD25^+^CD127^+^ subset also showed increased Bcl‐2 expression with Proleukin; however, the relative fold increase in Bcl‐2 expression by this effector subset was less compared to that observed for T_reg_.

## Discussion

IL‐2 is a crucial cytokine for survival and function of T cells, including T_reg_. We demonstrated in this study that very low dose clinical grade IL‐2 (Proleukin) induces STAT‐5 phosphorylation selectively in CD4^+^CD25^+^CD127^–^ T_reg_ from blood and liver of patients with AILD and is accompanied not only by a series of phenotypic and functional changes, but also up‐regulates Bcl‐2 to support T_reg_ survival. These data support existing evidence in autoimmune conditions such as diabetes and vasculitis that VLDIL‐2 administration contributes to the maintenance of self‐tolerance by increasing T_reg_ frequency 23, 25, 30. Our study extends the potential of VLDP therapy to the treatment of autoimmune liver diseases.

Administration of the IL‐2 or the IL‐2 : anti‐IL‐2 monoclonal antibody complex reduces autoimmune disease in rodent models 31, 32. Studies in vasculitis, graft‐*versus*‐host disease, systemic lupus erythematosus and type 1 diabetes suggest that an IL‐2 dose range of 0·3–3·0 × 10^6^ IU/m^2^ is well tolerated, and achieves a preferential increase in the percentage of T_reg_ with no induction of effector T cell activation 23, 25, 33, 34. The dose range we have identified working with clinical grade IL‐2 (Proleukin) *in vitro* is similar to other investigators, who have applied VLDP in autoimmune diseases 11, 23, 25, 34. High‐dose IL‐2 is approved in cancer therapy, where it promotes tumour killing by activating NK cells and CD4^+^ and CD8^+^ effector T cells 12; however, pulmonary vascular leakage occurs as a side effect to this treatment 35. By avoiding the activation of effector cells, VLDP should be safe for clinical application in general. Indeed, a former dose escalation study of low‐dose IL‐2 for treatment of graft‐*versus*‐host disease reported very few adverse events except skin induration in a minority of study patients. Other potentially linked rare side effects include fatigue, malaise, fever, thrombocytopenia and raised serum creatinine 23. Despite this, it is prudent that low‐dose Proleukin therapy in a hospital setting be restricted to patients with normal cardiac and pulmonary function.

IL‐2 is crucial for T_reg_ function. Binding of IL‐2 to IL‐2RA (CD25) on T_reg_ leads to phosphorylation of STAT‐5, resulting in up‐regulation of FoxP3 36 and functional surface markers. FoxP3 is essential for T_reg_ development and function, as evident from the occurrence of immunodysregulation polyendocrinopathy enteropathy X‐linked (IPEX) syndrome in patients with mutations in FoxP3 37, 38. Although T_reg_ do not secrete IL‐2, their high expression of CD25 allows them to consume and respond to low concentrations of exogenous IL‐2 39, 40. CD4^+^CD25^–^CD127^+^ effector T cells express intermediate‐affinity IL‐2 receptors, IL‐2RB and IL‐2RG constitutively; thus, they require higher concentrations of IL‐2 for activation in the absence of T cell receptor (TCR) engagement 41. Importantly, we observed that applying VLDP of less than 5 IU/ml could enhance FoxP3 selectively in T_reg_ compared to CD8^+^, CD4^+^CD25^–^CD127^+/–^ and CD4^+^CD25^+^CD127^+^ effector T cells from the blood and liver of patients with AILD. There was no noticeable activation of these effector subsets based on expression of CD69, granzyme B or 2B4.

CTLA‐4 is an essential functional marker on T_reg_ 42. It functions by removing its co‐stimulatory ligands, CD80/CD86, from antigen‐presenting cells by transendocytosis 43. Mice deficient in CTLA‐4 display an autoimmune phenotype and immune dysregulation is observed in patients with CTLA‐4 polymorphisms 44. Our data suggest that VLDP up‐regulates CTLA‐4 expression by T_reg_, but has no impact on CD4^+^CD25^+^CD127^+^ effector T cells. Furthermore, we identify that CTLA‐4 is a significant mediator in the mechanisms by which VLDP confers enhanced suppressive potential to T_reg_; VLDP also maintains T_reg_ functional markers such as GITR and OX40.

We have reported recently that the inflamed liver microenvironment is deficient in IL‐2 protein and that activated intrahepatic T cells are the main source of IL‐2 in the human liver 4. Local consumption of IL‐2 by immune cells, including T_reg_, creates a microenvironment that is deficient in IL‐2 to the level that it is unable to support T_reg_ function. This finding is consistent with our previous observation that only some of the FoxP3^+^ T_reg_ in the liver undergo STAT‐5 phosphorylation 45. We observed that liver T_reg_ had higher baseline expressions of CTLA‐4, CD25 and FoxP3 than peripheral T_reg_ and these were equivalent to levels seen on peripheral blood T_reg_ after IL‐2 stimulation. A higher baseline expression probably reflects the more activated, effector state of liver *versus* blood lymphocytes. Liver T_reg_ also expressed higher levels of the TNF receptor superfamily members CD137, OX40 and GITR than peripheral T_reg_, and comparing our current data in peripheral blood with our previous phenotyping of intrahepatic T_reg_ in AILD we identify that CD69 (40%), LAG3 (20%), Tim3 (5%) and cytolytic granzyme B (5%) are also all up‐regulated on T_reg_ in the liver compared to periphery (< 5% in peripheral blood) 4. This is in contrast to CD39 and PD‐1, which are expressed similarly on T_reg_ at both sites 4.

The effectiveness of T_reg_ function within the inflamed tissue setting might be compromised due to the action of inflammatory cytokines such as IL‐6, IL‐8, IL‐12, IFN‐γ and IL‐1βγ 4, which can enhance effector T cell activation and proliferation at the expense of T_reg_. Thus, exogenous IL‐2 such as VLDP might help to counteract these inflammatory signals 46, 47. CTLA‐4‐mediated T_reg_ suppressive function is via depletion of CD80/86 through transendocytosis 43. Excitingly, we observed that VLDP enhances T_reg_ suppressive function via CTLA‐4.

Recruitment of peripheral blood T_reg_ to the site of hepatic inflammation is dependent upon expression of the tissue‐homing CXCR3 chemokine receptor 45, 48, 49. We found expression of CXCR3 on approximately 50% of peripheral blood T_reg_ from AILD patients, comparable to controls, suggesting that cells from patients, even on immunosuppression, retain the liver‐homing receptors for recruitment. Moreover, we report that CXCR3 expression was unaffected by VLDP, implying that T_reg_ of patients on VLDP therapy would retain the capability to home to inflamed hepatic lobules and portal tracts. We have reported recently that human liver‐infiltrating T_reg_ are mainly of CD45RA^–^CCR7^–^ phenotype 4. Our findings now demonstrate that exogenous VLDIL‐2 up‐regulates T_reg_ functional markers predominantly in this effector (suppressor) population in all types of AILD. A recent study also described high expression of CXCR3 in CD4^+^ T cells in early‐stage PBC, which was associated with increased demethylation of the CXCR3 promoter. This up‐regulation was most striking in the activated memory CD45RO^+^ population 50. Taken together, these data suggest that CD4^+^ T_reg_ from patients with AILD will recruit to the human liver and be potentiated functionally by IL‐2 therapy.

T_reg_ depend upon IL‐2 for expansion and survival and cells undergo apoptosis upon IL‐2 deprivation 51. Bcl‐2 is a critical target in IL‐2 signalling 18, 52, helping to protect responding cells from apoptosis. A recent study also confirmed that use of a pan‐Bcl‐2 inhibitor leads to profound down‐regulation of FoxP3 and CTLA‐4 and a reduction in the suppressive function of T_reg_ 53. We demonstrated that VLDP enhances Bcl‐2 expression selectively in T_reg_ but not CD8^+^ T cells or CD4^+^ T effector cells from both peripheral blood and liver. This is an important observation because enhanced survival of immune‐regulatory T_reg_ is critical to the maintenance of tolerance.

In conclusion, our findings provide compelling evidence to support the design of T_reg_‐directed Phase I and II clinical trials administering VLDP as a cytokine monotherapy or in combination with autologous T_reg_ cell therapy in AILD. Given its short half‐life, regular doses of VLDP may be required to maintain efficacy.

## Author contributions

Y. H. O. and H. C. J. designed the experiments. H. C. J. L. E. J. and P. L. performed the experiments, analysed the data and prepared the figures. H. C. J., L. E. J., P. L., D. H. A. and Y. H. O. wrote the manuscript. G. W., M. C. and G. H. consented patients and collected clinical data. All authors reviewed the manuscript.

## Disclosure

Y. H. O and H. C. J. are funded by Clinician Scientist Award from the Medical Research Council, Queen Elizabeth Hospital Charity and National Institute for Health Research Liver Biomedical Research Unit, Birmingham. All authors declare that there are no financial conflicts of interest associated with this work. This paper presents independent research supported by the Birmingham NIHR Liver Biomedical Research Unit based at the University of Birmingham and University Hospitals Birmingham NHS Foundation Trust. The views expressed are those of the authors and not necessarily those of the NHS, the NIHR or the Department of Health.

## Supporting information

Additional Supporting Information may be found in the online version of this article at the publisher's website.

**Fig. S1.** Flow cytometry gating strategy for the analysis of signal transducer and activator of transcription‐5 (STAT‐5) expression by regulatory T cells (T_reg_) and eight other immune cell subsets.
**Fig. S2.** Very low dose interleukin (IL)−2 selectively up‐regulates signal transducer and activator of transcription‐5 (STAT‐5) in peripheral regulatory T cells (T_reg_) from autoimmune hepatitis (AIH) and primary biliary cholangitis (PBC) patient bloods. Peripheral blood mononuclear cells (PBMCs) from patients with AIH, PBC or primary sclerosing cholangitis (PSC) were stimulated for 10 min with IL‐2 (Proleukin) 0‐100 IU/ml and the percentage expression of phosphorylated (Y694)STAT5 by each leucocyte population assessed by flow cytometry at each IL‐2 dose. Data are mean ± standard error of the mean (s.e.m.) for five donors (AIH remission), four donors (AIH relapse) and three donors (PBC and PSC).
**Fig. S3.** Flow cytometry gating strategy (a) to define CD4, CD8 and regulatory T cell (T_reg_) populations and representative overlays (b,d) for markers of T cell activation and function on CD4, CD8 and T_reg_ in autoimmune liver disease (AILD) blood [autoimmune hepatitis (AIH)] and liver [primary sclerosing cholangitis (PSC)] following 18 h exposure to Proleukin 5 IU/ml.
**Fig. S4.** Effect of very low dose interleukin (IL)−2 on expression of IL‐2‐regulated regulatory T cell (T_reg_) functional markers CD25, cytotoxic T lymphocyte antigen‐4 (CTLA‐4) and forkhead box protein 3 (FoxP3^+^) by CD4, CD8 and T_reg_ cells from blood and liver. Peripheral blood mononuclear cells (PBMCs) from patients with autoimmune hepatitis (AIH), primary sclerosing cholangitis (PSC) and primary biliary cholangitis (PBC) and liver infiltrating lymphocytes from autoimmune liver disease (AILD) livers were exposed to 0 or 5 IU/ml IL‐2 (Proleukin) for 18 h and the percentage expression of CD25, CTLA‐4 and FoxP3 by CD4^+^, CD8^+^ and T_reg_ cells examined by flow cytometry. Data are mean ± standard error of the mean (s.e.m.)for five donors (AIH), three donors (PSC) and five donors (liver).
**Fig. S5.** Sustained exposure to low dose interleukin (IL)−2 up‐regulates signal transducer and activator of transcription‐5 (STAT‐5) selectively in CD4^+^CD25^+^CD127^+^ T cells as well as in regulatory T cells (T_reg_). Peripheral blood mononuclear cells (PBMCs) from patients with autoimmune liver disease (AILD) were stimulated for 18 h with IL‐2 (Proleukin) doses of 0, 1, 5 and 10 IU/ml and expression of phosphorylated (Y694)STAT‐5 by each leucocyte population assessed by flow cytometry. (a) Percentage of each leucocyte population expressing pSTAT‐5 at each IL‐2 dose. (b) median fluorescence intensity (MFI) for pSTAT‐5.
**Fig. S6.** Effect of very low dose interleukin (IL)−2 on the suppressive ability of regulatory T cells (T_reg_) in autoimmune liver diseases. (a) CD4^+^CD25^–^ T responder cells were isolated from peripheral blood mononuclear cells (PBMCs) of controls (*n* = 3) and the effect of blocking CD28‐mediated co‐stimulation with Abatacept [cytotoxic T lymphocyte antigen‐4‐immunoglobulin (CTLA‐4‐Ig)] on division index at 5 days examined. (B) CD4^+^CD25^+^CD127^–^ T_reg_ and autologous CD4^+^CD25^–^ T responder cells were isolated from PBMC of two patients with autoimmune hepatitis (AIH) (one in remission and one in relapse). T responders were labelled with cell trace violet, and following overnight exposure of T_reg_ to 0 or 5 IU/ml Proleukin were co‐cultured with the T_reg_ in the presence of anti‐CD3 and dendritic cells, with or without CTLA‐4 blockade. Cell trace violet dilution indicating T responder cell division was analysed by flow cytometry at 5 days.
