# Supplementary material for: Low‐dose interleukin‐2 promotes STAT‐5 phosphorylation, Treg survival and CTLA‐4‐dependent function in autoimmune liver diseases
Source: Clin Exp Immunol. 2017 Mar 20;188(3):394–411. doi: 10.1111/cei.12940 (PMC5422719; doi:10.1111/cei.12940)
Supplement: Supplementary file 1 — Fig. S1. Flow cytometry gating strategy for the analysis of signal transducer and activator of transcription‐5 (STAT‐5) expression by regulatory T cells (Treg) and eight other immune cell subsets. Fig. S2. Very low dose interleukin (IL)−2 selectively up‐regulates signal transducer and activator of transcription‐5 (STAT‐5) in peripheral regulatory T cells (Treg) from autoimmune hepatitis (AIH) and primary biliary cholangitis (PBC) patient bloods. Peripheral blood mononuclear cells (PBMCs) from patients with AIH, PBC or primary sclerosing cholangitis (PSC) were stimulated for 10 min with IL‐2 (Proleukin) 0‐100 IU/ml and the percentage expression of phosphorylated (Y694)STAT5 by each leucocyte population assessed by flow cytometry at each IL‐2 dose. Data are mean ± standard error of the mean (s.e.m.) for five donors (AIH remission), four donors (AIH relapse) and three donors (PBC and PSC). Fig. S3. Flow cytometry gating strategy (a) to define CD4, CD8 and regulatory T cell (Treg) populations and representative overlays (b,d) for markers of T cell activation and function on CD4, CD8 and Treg in autoimmune liver disease (AILD) blood [autoimmune hepatitis (AIH)] and liver [primary sclerosing cholangitis (PSC)] following 18 h exposure to Proleukin 5 IU/ml. Fig. S4. Effect of very low dose interleukin (IL)−2 on expression of IL‐2‐regulated regulatory T cell (Treg) functional markers CD25, cytotoxic T lymphocyte antigen‐4 (CTLA‐4) and forkhead box protein 3 (FoxP3+) by CD4, CD8 and Treg cells from blood and liver. Peripheral blood mononuclear cells (PBMCs) from patients with autoimmune hepatitis (AIH), primary sclerosing cholangitis (PSC) and primary biliary cholangitis (PBC) and liver infiltrating lymphocytes from autoimmune liver disease (AILD) livers were exposed to 0 or 5 IU/ml IL‐2 (Proleukin) for 18 h and the percentage expression of CD25, CTLA‐4 and FoxP3 by CD4+, CD8+ and Treg cells examined by flow cytometry. Data are mean ± standard error of the mean (s.e.m.)for fi [file CEI-188-394-s001.pdf]

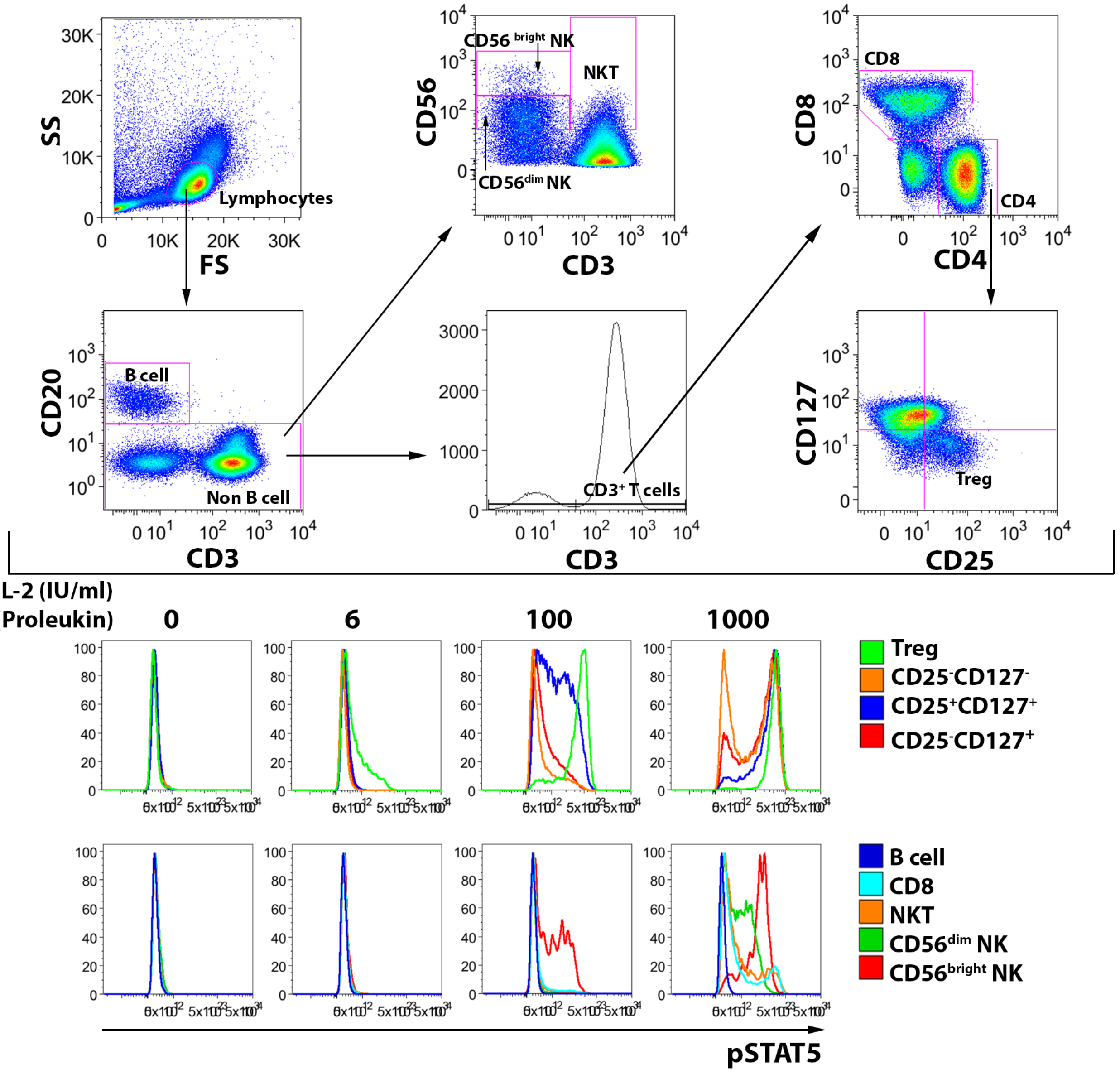

SS
FS
Lymphocytes
CD56
CD3
CD56 bright NK
NKT
CD56dim NK
CD8
CD4
CD20
B cell
Non B cell
CD3+ T cells
CD127
CD25
Treg
IL-2 (IU/ml)
(Proleukin)
0
6
100
1000
Treg
CD25-CD127-
CD25+CD127+
CD25-CD127+
B cell
CD8
NKT
CD56dim NK
CD56bright NK
pSTAT5

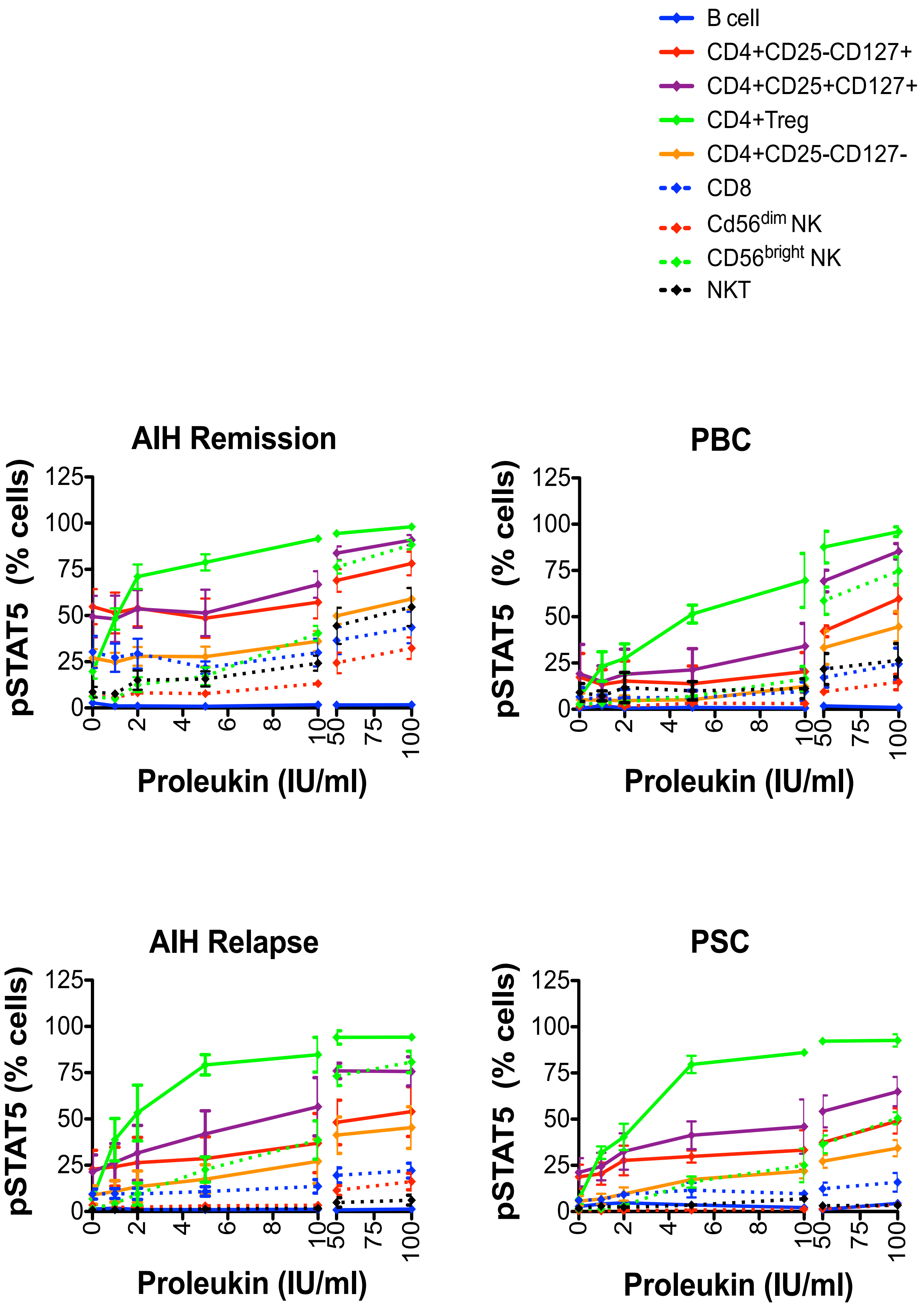

B cell
CD4+CD25-CD127+
CD4+CD25+CD127+
CD4+Treg
CD4+CD25-CD127-
CD8
Cd56dim NK
CD56bright NK
NKT
AIH Remission
PBC
AIH Relapse
PSC
pSTAT5 (% cells)
Proleukin (IU/ml)
0
25
50
75
100
125
0
2
4
6
8
10
50
75
100

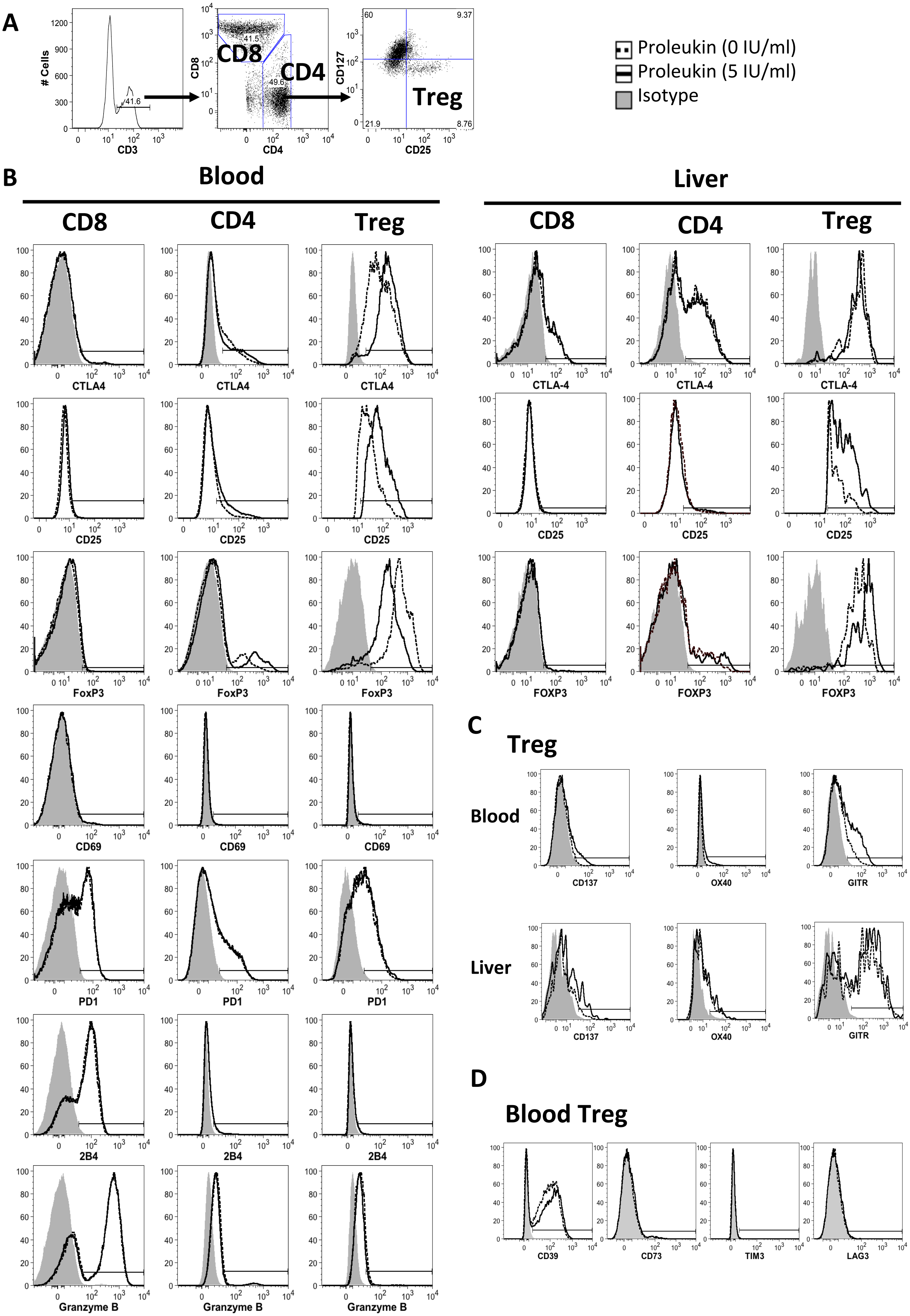

A
Cells
CD3
41.6
CD8
CD4
41.5
49.6
CD8
CD4
CD127
CD25
60
9.37
21.9
8.76
Treg
Proleukin (0 IU/ml)
Proleukin (5 IU/ml)
Isotype
B
Blood
Liver
CD8
CD4
Treg
CTLA4
CTLA-4
CD25
FoxP3
FOXP3
CD69
PD1
2B4
Granzyme B
C
Treg
Blood
Liver
CD137
OX40
GITR
D
Blood Treg
CD39
CD73
TIM3
LAG3

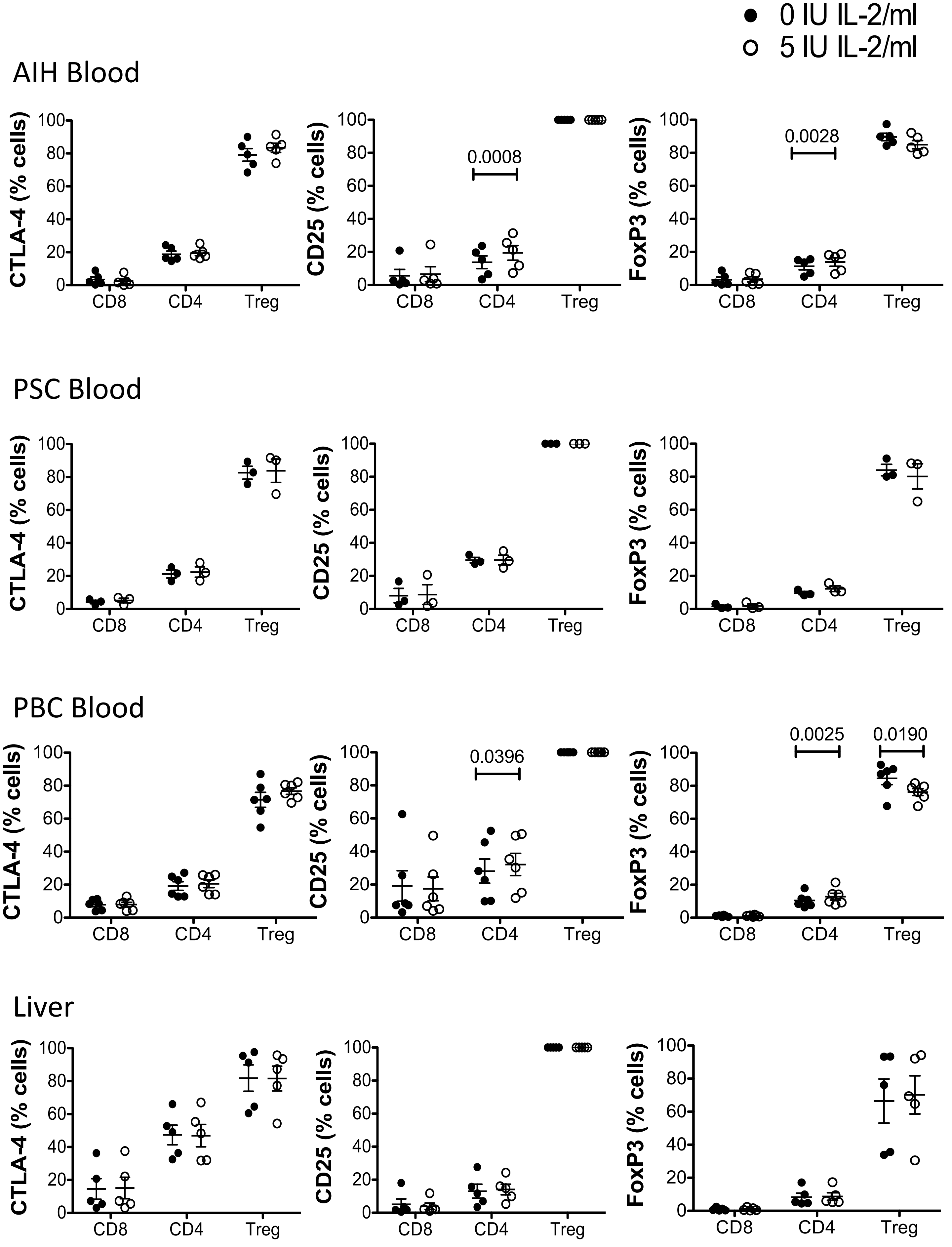
0 IU IL-2/ml
5 IU IL-2/ml
AIH Blood
PSC Blood
PBC Blood
Liver
CTLA-4 (% cells)
CD25 (% cells)
FoxP3 (% cells)
CD8
CD4
Treg
0.0008
0.0028
0.0396
0.0025
0.0190

(A)

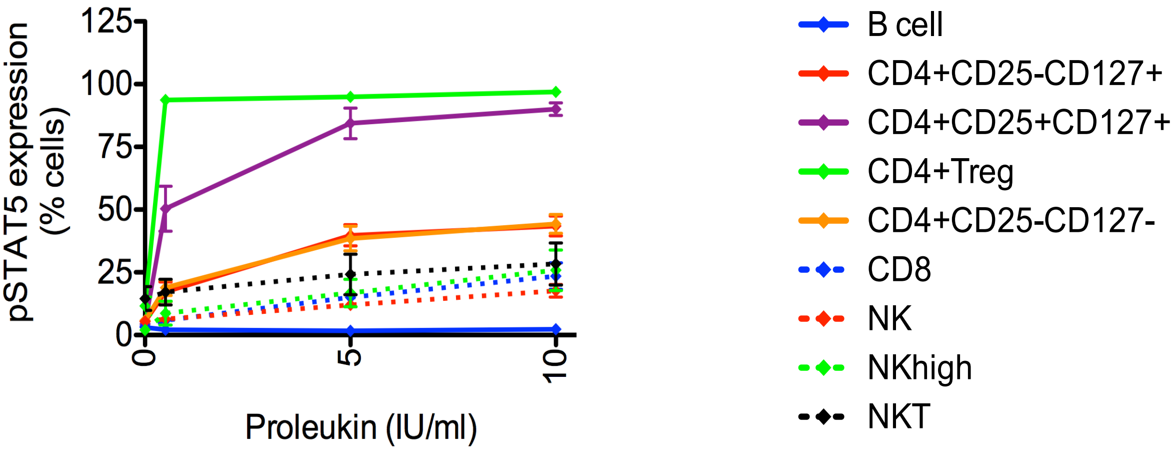

pSTAT5 expression (% cells)
125
100
75
50
25
0
0
5
10
Proleukin (IU/ml)
B cell
CD4+CD25-CD127+
CD4+CD25+CD127+
CD4+Treg
CD4+CD25-CD127-
CD8
NK
NKhigh
NKT

(B)

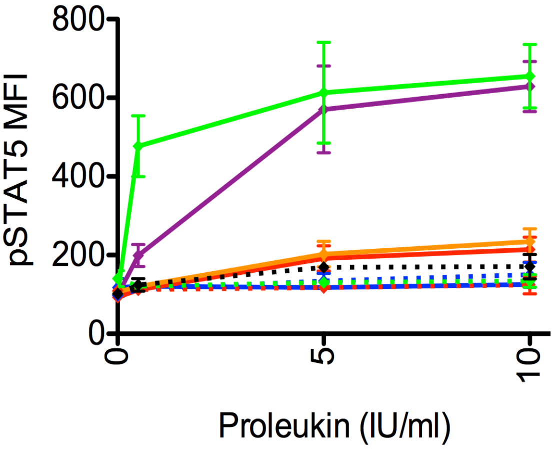

pSTAT5 MFI
800
600
400
200
0
0
5
10
Proleukin (IU/ml)

(A)

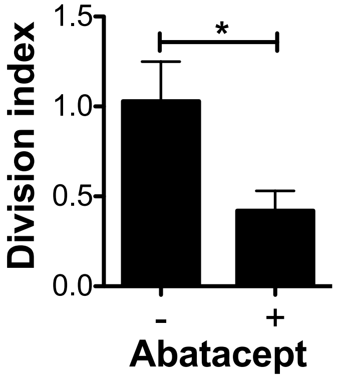
Division index
1.5
1.0
0.5
0.0
*
-
+
Abatacept

(B)

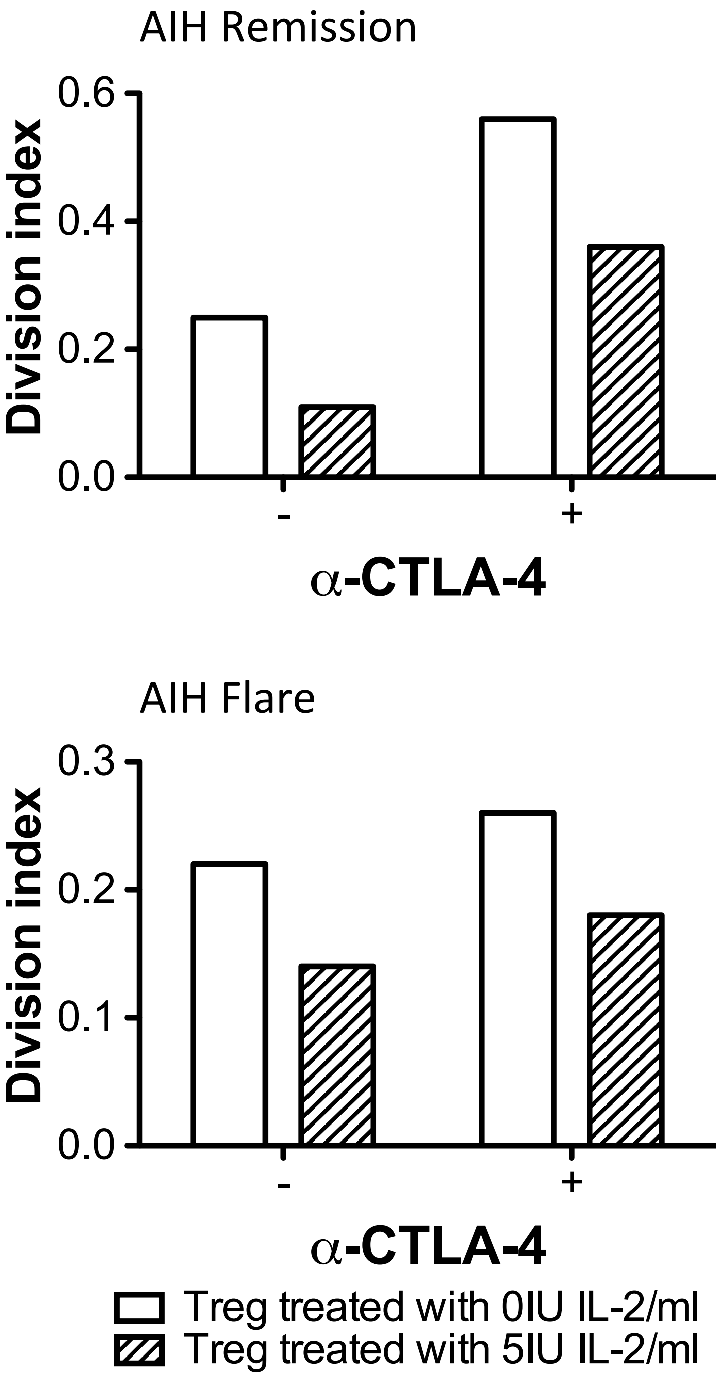
AIH Remission
Division index
0.6
0.4
0.2
0.0
-
+
α-CTLA-4
AIH Flare
Division index
0.3
0.2
0.1
0.0
-
+
α-CTLA-4
Treg treated with 0IU IL-2/ml
Treg treated with 5IU IL-2/ml
